# An unexpected response to adenosine

**DOI:** 10.1007/s12471-014-0632-y

**Published:** 2014-11-19

**Authors:** J. M. ter Maaten, R. G. Tieleman

**Affiliations:** Department of Cardiology, Martini Hospital Groningen, van Swietenplein 1, 9728 NT Groningen, the Netherlands

Rhythm puzzle—answer

**Fig. 1:**
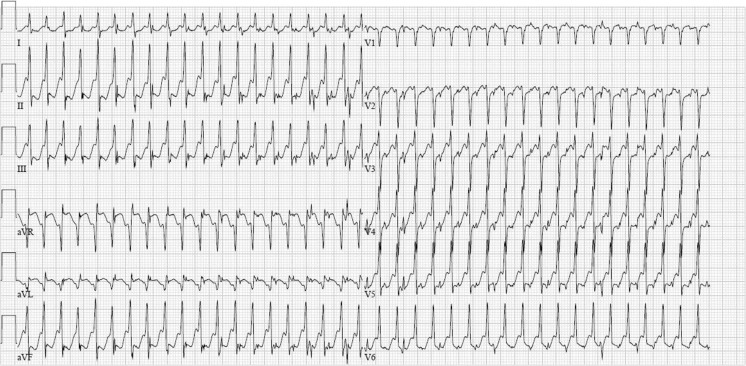
ECG after administration of adenosine

The ECG shown in Fig. 1 is a narrow complex tachycardia with a frequency of 270 beats/min. Contradictory to expectations, the administration of adenosine resulted not in conversion of the arrhythmia, but in this accelerated rhythm, making an AV(N)RT unlikely. Neither did it slow down the ventricular response rate, as would be expected during atrial tachycardia or atrial flutter. Several case reports have been described where adenosine caused an accelerated AV conduction during atrial flutter, leading to 1:1 conduction.[1] This is thought to be a result of increased sympathetic tone, caused by a direct effect of adenosine on the afferent sympathetic system.[2] On the other hand, as adenosine blocks the AV node, this can cause preferential conduction of an atrial tachycardia or atrial flutter over an accessory pathway, resulting in an accelerated ventricular response.[3] However, since the QRS of the resultant tachycardia remains narrow this must be an atria-Hisian bypass tract (Lown-Ganong-Levine syndrome).

The patient was referred for electrophysiological treatment of the flutter. No accessory bundle was identified. An atrial flutter circuit was ablated. The most likely explanation of the ECG shown in Fig. 1 is an atrial flutter with 1:1 atrioventricular conduction caused by increased sympathetic tone after administration of adenosine.
